# Updating ‘Data on the determination of human epidermis integrity in skin permeation experiments by electrical resistance’ with Data on pig ear skin

**DOI:** 10.1016/j.dib.2024.110295

**Published:** 2024-03-05

**Authors:** Paola Volontè, Umberto M. Musazzi, Chiara G.M. Gennari, Antonella Casiraghi, Francesco Cilurzo, Paola Minghetti

**Affiliations:** Department of Pharmaceutical Sciences, Università degli Studi di Milano, via G. Colombo 71, Milan 20133, Italy

**Keywords:** In vitro permeation, Franz's diffusion cells, Membrane integrity, Caffeine, Benzoic acid, Membrane model

## Abstract

The data presented in this article are an update of the dataset provided by Musazzi et al. [1] and are related to the research article entitled “Equivalence assessment of creams with quali-quantitative differences in light of the EMA and FDA regulatory framework” [2]. In vitro permeation study (IVPT) is typically conducted using the method of Franz's diffusion cell for assessing the biopharmaceutical performance of topically applied products. While the human epidermis is considered the benchmark, various animal models (for instance, pig ear) have been accepted as a permeation membrane. Nonetheless, it is crucial to evaluate the integrity of the membrane to ensure the quality of the experiments. The methods employed for this assessment vary, and the outcomes are heavily reliant on the operational conditions, and the model membrane. The article contributes to the existing dataset by providing data on the electrical resistance values of pig ear skin samples and their correlation with the in vitro permeability fluxes of caffeine and benzoic acid. This data is utilized to determine a suitable cut-off for verifying the skin integrity of such an animal model. This information could be beneficial for facilitating critical or comprehensive analyses, contributing to the creation of a standard method.

Specifications Table

**Table:** 

Subject	Pharmaceutical Sciences
Specific subject area	Evaluation of skin integrity for in vitro permeation tests
Type of data	Figs., Text File. Raw, Analysed.
Data collection	Electrical resistance measurement (Agilent 4263B LCR Meter, Microlease, I), Franz's diffusion cells (PermeGear, US)
Data source location	University of Milan, Milan, Italy
Data accessibility	Repository name: Electrical resistance and permeation fluxes through pig ear skin Data identification number: 10.17632/kjxjk4×88s.2 Direct URL to data: https://data.mendeley.com/datasets/kjxjk4×88s/2
Related data article	[1] U.M. Musazzi, A. Casiraghi, S. Franzé, F. Cilurzo, P. Minghetti. Data on the determination of human epidermis integrity in skin permeation experiments by electrical resistance. Data Brief, 2018, 26;21:1258-1262.
Related research article	[2] P. Volontè, U.M. Musazzi, L. Arnaboldi, M.A. Ortenzi, A. Casiraghi, F. Cilurzo, P. Minghetti. Equivalence assessment of creams with quali-quantitative differences in light of the EMA and FDA regulatory framework. European Journal of Pharmaceutical Sciences, 2024, 195, 106726, doi:10.1016/j.ejps.2024.106726

## 1 Value of the Data

• The data adds value to the existing dataset by providing evidence of the correlation between electrical resistance and permeation flux through an animal model of skin membrane. The choice of skin membrane model and the assessment of its integrity are crucial steps in the validation of IVPT protocols for topically applied products. Although the human epidermis is the gold standard, other animal models have been accepted. However, inter-laboratory comparability of IVPT data is limited due to the lack of standards on acceptable range, methods, and experimental conditions.
• The dataset can be useful for developing an inter-laboratory method based on electrical resistance to assess membrane integrity when pig-ear skin is used for in vitro permeation studies. The data allowed for the definition of a cut-off (10 kΩcm2) for electrical resistance measurements (voltage at 100 mV and frequency at 100 Hz) to select pig-ear epidermis samples with acceptable permeability characteristics for in vitro permeation studies using Franz's diffusion cells.

## 2 Background

The inclusion of in vitro human skin permeation studies (IVPT) in regulatory guidelines [3] paves the way for new possibilities in the development of topically applied dosage forms and their follow-on products, allowing the waiver and/or optimization of supportive in vivo trials. However, the experimental setup must be clearly designed and validated, which includes the choice of skin membrane model and the assessment of its integrity. The use of ex vivo viable human epidermis is the gold standard for IVPT, while cadaver skin can be a feasible alternative if the national regulatory framework allow its use. Alternately, other animal models (e.g., pig ear skin) have been accepted as alternatives [4]. Even though the control of skin integrity is a standard procedure typically carried out prior to each permeation experiment, the existing data on acceptable ranges are still scarce and/or vary based on the model membrane, methods, and experimental conditions [5]. As a result, inter-laboratory comparability of IVPT data remains challenging. Previously, data on IVPT through human epidermis contribute to fill the gap on correlation between electrical resistance of human epidermis and permeation fluxes of model permeants [1]. This article upgrades such an existing dataset, providing data obtained using the same methodological approach, but using pig ear skin as a membrane.

## 3 Data Description

This article describes the dataset of electrical resistance value and permeation fluxes collected during IVPT using pig-ear skin as model of skin membrane [6]. It consists of two series of data obtained from IVPT conducted using 0.1% of caffeine (*n =* 26) and 0.3% of benzoic acid solution (*n =* 26) as a permeant. For each series of experiment, both value of electrical resistance of membrane specimens and permeation flux of drug (i.e., benzoic acid, caffeine) were collected following the same methodological approach described in the existing dataset [1].

**Fig. 1:**
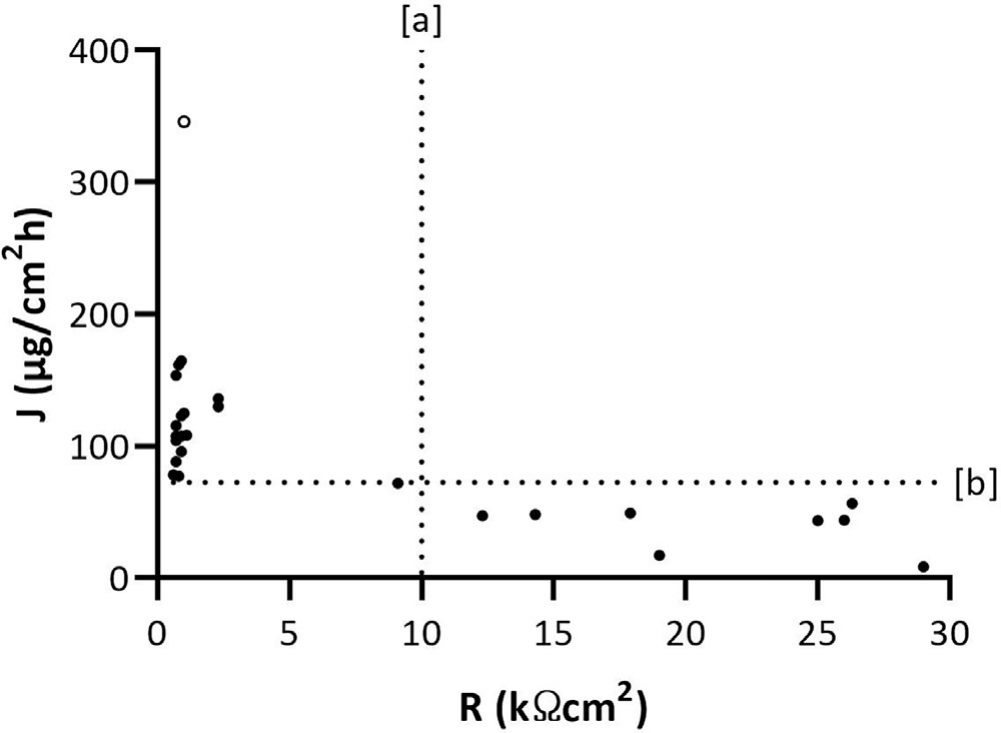
Electrical resistance (R) and permeation data of benzoic acid obtained by in vitro permeation studies performed using 26 pig-ear skin donors. [a] cut-off of electrical resistance set-up for permeation studies through pig-ear skin; [b] Maximal value (i.e., 72.56 µg/cm^2^h) of acceptance range for benzoic acid permeation fluxes in the existing dataset [1]. Outlier based on Dixon’s test is represented with an open circle in the graph.

**Fig. 2:**
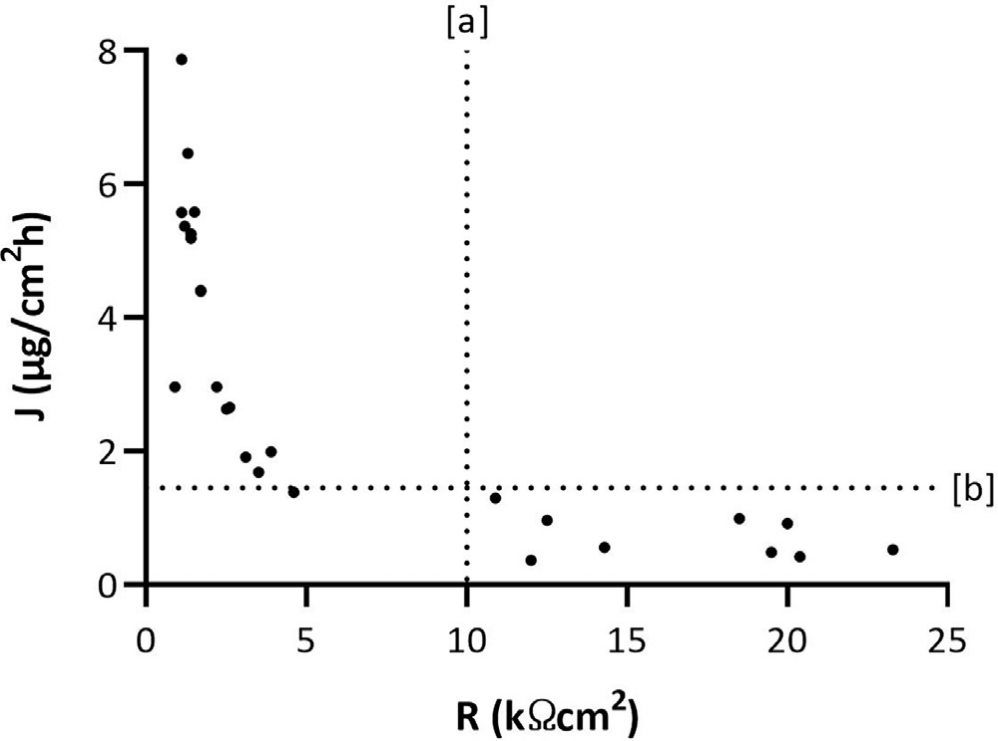
Electrical resistance (R) and permeation data of caffeine obtained by in vitro permeation studies performed using 26 pig-ear skin donors. [a] cut-off of electrical resistance set-up for permeation studies through pig-ear skin; [b] Maximal value (i.e., 1.45 µg/cm^2^h) of acceptance range for caffeine permeation fluxes in dataset reported in the existing dataset [1]. No outliers identified based on Dixon's test.

Figs. 1 and 2 showed permeation fluxes of benzoic acid and caffeine plotted versus electrical resistance, respectively. The electrical resistance of pig-ear skin specimens (median: 2.25 kΩcm^2^; Interquartile Range: 0.98 – 13.85 kΩcm^2^) agrees with previously published data [7]. Overall data showed that the electrical resistance, and therefore the skin integrity, more critical for skin permeation of poorly permeable compounds (i.e., caffeine) than other permeants (e.g., benzoic acid). J-values of both benzoic acid and caffeine were in the same order of magnitude of data provided previously in the existing dataset [1,6].

Data demonstrated that the same methodological approach applied for validating IVPT through human epidermis [1,5] is sensitive enough to assess the integrity of pig-ear skin. The cut-off value of electrical resistance can be extrapolated from pig-ear skin specimen showing a permeation flux of benzoic acid or caffeine falls in the same range determined by IVPT through human epidermis. The maximal J-values reported in existing dataset for benzoic acid and caffeine are 72.56 µg/cm^2^h and 1.45 µg/cm^2^h, respectively [1]. Therefore, based on novel data provided from pig-ear skin specimens, a cut-off value of 10 kΩcm^2^ can be identify and applied in further IVPT to discard specimens having lower electrical resistance.

## 4 Experimental Design, Materials and Methods

### 4.1 Skin permeation studies of benzoic acid and caffeine

The porcine ears were provided by a local slaughterhouse. All experiments were performed on frozen and thawed skin, stored at -20°C and used for the experiments within 6 months. The part of skin required for the experiments was removed from the outer side of the pig's ear, cut and dermatomized using dermatome (de Soutter Medical, Nieuwegein, Netherlands). The thickness of the skin piece was measured with an external digital electronic micrometer (model MI-1000, ChemInstruments, Fairfield, Ohio, USA) and samples considered for the permeation experiments had a thickness between 0.630 and 0.770 mm. For each series of experiment, twenty-six pig-ear skin specimens obtained from different pig donors were mounted on Franz's diffusion cells (permeation area: 0.636 cm^2^; receptor volume: ≈3 mL, PermeGear, US), whose receptor compartments were filled with 0.9% w/v saline solution. At the beginning of the experiment R-value was measured by the instrument Agilent 4263B LCR Meter (Microlease, Italy) equipped with Ag/AgCl electrodes. One electrode was inserted into the receptor compartment from the cell sampling channel, whereas the second one was inserted directly into the donor compartment. In-house designed Teflon® supports were used to maintain electrode in the correct position throughout the experiment. The voltage and the frequency were set at 100 mV and at 100 Hz, respectively. Then, the donor solution was withdrawn and replaced with 0.5 mL of 0.1% caffeine (series A) or 0.3 % benzoic acid solutions (series B). The membrane surface temperature was kept at 32 ± 1°C throughout the experiments. The in vitro permeation studies were performed using the same experimental protocol reported by Volontè et al. [2]. Briefly, at predetermined times (1, 3, 5, 7, 24 h), 200 µL samples were withdrawn from the receptor compartment and replaced with a fresh receptor phase. Sink conditions were maintained throughout the experiments. Samples were analysed by HPLC according to the methods described below. The permeation flux (J) was determined as the slope of the linear portion of the plot of the cumulative amount permeated through the skin per unit area versus time. The possible outliners within each series were checked by Dixon's Q test (Q = 95%).

### 4.2 HPLC methods

The amount of benzoic acid and caffeine was determined by high performance liquid chromatography (HPLC; HP 1100 ChemStations, Agilent Technologies, Santa Clara, US), equipped with ultraviolet detector. ***Benzoic acid***: UV wavelength was set at 235 nm. Water/acetonitrile/acetic acid (65/35/1) was used as mobile phase at a flow rate of 1.0 mL/min and the analysis temperature was fixed at 25°C. Compound separation was carried out using reverse-phase column (InertClone ODS, 250×4.6 mm, 5 µm, Phenomenex Inc., USA) and the injection volume was set at 20 µL. Drug concentration was determined from two standard calibration curves in the range 0.1–500 µg/mL. ***Caffeine***: UV wavelength was set at 272 nm. Water/acetonitrile/acetic acid (90/10/1) was used as mobile phase at a flow rate of 1.2 mL/min and the analysis temperature was fixed at 25°C. Compound separation was carried out using reverse-phase column (InertClone ODS, 150×4.6 mm, 5 µm, Phenomenex Inc., USA) and injection volume was set at 20 µL. Drug concentration was determined from two standard calibration curves in the range 0.01–100 µg/mL.

## Limitations

The primary limitations of the dataset are related to its limited size. Moreover, the relevance of provided dataset should be evaluated taking into consideration the possible inter-laboratory variability in terms of animal source, membrane thickness, and methodological set up.

## Ethics Statement

The authors have adhered to the ethical requirements for publication in Data in Brief. They confirm that the current work does not involve human subjects, animal experiments, or any data collected from social media platforms.

## CRediT authorship contribution statement

**Paola Volontè:** Investigation. **Umberto M. Musazzi:** Conceptualization, Methodology, Writing – original draft. **Chiara G.M. Gennari:** Data curation. **Antonella Casiraghi:** Methodology. **Francesco Cilurzo:** Writing – review & editing. **Paola Minghetti:** Supervision.

## Data Availability

Electrical resistance and permeation fluxes through pig ear skin (Original data) (Mendeley Data)
